# Investigation of *Waltheria indica* L. (sleepy morning plant) as pathogen killing agent from shallow well water revealed a potential alternative for water disinfection

**DOI:** 10.1371/journal.pone.0330987

**Published:** 2025-09-04

**Authors:** Josephat Ilunde, Nelson Mpumi, Mwema Felix Mwema, Revocatus L. Machunda

**Affiliations:** School of Materials, Energy, Water and Environmental Sciences (MEWES), The Nelson Mandela African Institution of Science and Technology (NM-AIST), Arusha, Tanzania; Universidad Autonoma de Chihuahua, MEXICO

## Abstract

In many parts of the world, shallow well drinking water is contaminated and often consumed without treatment, contributing to over 3.4 million deaths annually due to water-related diseases. This research aims to assess the efficacy of *Waltheria indica* plant root extracts as an affordable method for disinfecting shallow well water in rural Tanzania. Laboratory experiments were conducted using dried plant roots in powdered form, as well as soaked and boiled extracts, to test their effectiveness in eliminating *Escherichia coli* and *total coliforms* at varying concentrations and contact times. Contaminated water samples were collected from shallow wells in the Misungwi district, Tanzania, and treated using all three forms of *Waltheria indica* extracts. Phytochemical profiling of the plant root extracts was performed using Liquid Chromatography tandem Mass Spectrometry (LC-MS/MS) to identify potential antimicrobial compounds. The disinfection efficacy was evaluated using a flocculator with 1-litre beakers to determine bacterial removal and the effects on key physicochemical parameters. Results showed that all three extract forms significantly reduced *E. coli* and *total coliforms*, achieving up to 100% reduction within 12 hours. The soaked extract was most effective, reducing *E. coli* and *total coliforms* by approximately 94.3% and 93.8%, respectively, followed by the boiled extract (83.9% and 85.8%) and the powdered form (81.9% and 68.2%). The treated water’s pH and total dissolved solids (TDS) were not significantly affected across all treatments. However, turbidity levels remained above the acceptable limit of 5 NTU, indicating the need for further optimization. Overall, the results suggest that *Waltheria indica* plant root extracts have strong potential as a natural, low-cost water disinfectant.

## 1. Introduction

In sub-Saharan Africa, the right of accessibility to clean and safe drinking water remains unfulfilled due to climate change leading to drought and drying of water sources. The establishment of enough shallow well water for drinking and other domestic uses as a means of mitigating water scarcity has become an alternative source in many African and sub-Saharan countries [1]. However, shallow well water sources in sub-Saharan Africa are susceptible to contamination by pathogens from animal and human excreta, thus compromising water quality and leading to various waterborne diseases [2]. Outbreaks of waterborne diseases in sub-Saharan Africa resulting from shallow well water contamination have triggered numerous health crises, leading to ailments such as diarrhoea and gastroenteritis diseases and, in more serious cases, fatalities caused by various bacterial, viral, and protozoan pathogens such as *Salmonella typhimurium*, *Escherichia coli, Candida albicans*, *Vibrio cholera, Norovirus* and *Entamoeba histolytica* [2–4].

Thus, the region is severely affected, with millions of people falling victim to waterborne diseases such as diarrhoea, cryptosporidiosis, cholera, and typhoid fever [5,6]. It is a harsh reality that every year, 3.4 million people, predominantly children and the elderly, succumb to water-related illnesses [7].

In Misungwi district, Tanzania, particularly, stomach-related health issues such as diarrhoea, schistosomiasis, typhoid, and intestinal worm infections are very common and widespread. Those water-borne diseases affect human health. Even though many water-borne diseases infect humans, the most infectious disease is gastroenteritis, which causes diarrhoea and dysentery. This might be due to the presence of *Escherichia coli* and *total coliforms,* which could be linked to the consumption of animals and human excreta from shallow well contaminated water [8].

Currently, numerous methods have been employed to treat both shallow wells contaminated water, including coagulation, flocculation, and filtration worldwide and in Misungwi district and Tanzania generally. Moreover, technologies such as container storage, pot chlorination, ozonation, UV radiation, and boiling have been employed to disinfect shallow well drinking water [9–11]. However, many of these approaches rely on chemical disinfectants, such as calcium hypochlorite, which is widely recognized for its effectiveness [12] and require specialized expertise, such as water boiling, which may not always be sustainable, especially in situations where the supply of fuel for heating is lacking, or when charcoal is costly or unavailable [13]. These treatment methods are expensive to implement in most rural communities in Tanzania. As a result, most rural Tanzanians may not be able to afford the widespread use of existing physical and chemical disinfection methods for shallow wells treatment of water for domestic settings, particularly where economic disparities can be significant. Despite existing treatment methods, there remains a need for an alternative and appropriate approach to treat shallow well drinking water in rural Tanzania that effectively eliminates pathogens such as *Escherichia coli* and *total coliforms* to safeguard human health.

The people of Sub-Saharan Africa are blessed with a rich diversity of medicinal plant species, many of which contain bioactive compounds that have been traditionally used as health-promoting spices. These plants also hold potential for application in purifying and disinfecting shallow well water. For instance, dried beans (*Vicia faba*) and peach seeds (*Prunus vulgaris*) have been identified as potent natural bio-coagulants for groundwater purification with minimal to no adverse effects on human health when consumed [14]. Natural coagulants are particularly effective in removing turbidity from surface water [15]. Furthermore, the *Moringa oleifera* extract has been tested for removing pathogens in *faecal coliform-*contaminated drinking water, reducing the pathogen load and performing sufficiently according to the World Health Organization’s (WHO) 2014 guideline, i.e., zero CFU/100 mL for treated water [7]. Also, other plant extracts like *Strychnos potatorum* have been used as water purifier bio-coagulants, eliminating waterborne microbes from surface water [16]. The spice plant *Syzygium aromaticum* L. (also known as clove oil) has been tested under varying conditions and shown to exhibit antimicrobial activities, killing pathogens such as *Escherichia coli* and *Staphylococcus aureus*, which cause foodborne diseases [17,18]. Ginger (*Zingiber officinale*) has been studied for its antibacterial properties, which are attributed to its aromatic compounds, particularly phenolic compounds [19]. These compounds have demonstrated effective antibacterial activity against *E.coli* and *Staphylococcus aureus*, particularly in the preservation of food and beverages. [20]. Moreover, the black pepper (*Piper nigrum* L.) has been tested to possess an alluring aroma [21] and has potential bioactive compounds such as alkaloids, tannins, essential oils, saponins, and piperine [22], which are responsible for restricting bacterial activities, particularly *Escherichia coli* [23]. Black Pepper Essential Oil (BPEO) has the tendency to dislocate the bacteria cell structures to cause water permeability and finally die by lysis [24]. The roots of *Waltheria indica* L., a spicy plant known for its aromatic compounds, have not yet been evaluated for their ability to eliminate *Escherichia coli* and *total coliforms* from shallow well drinking water. Therefore, this study investigated the *Waltheria indica* L. plant roots as potential pathogen-killing agents in shallow well-drinking water from rural Tanzania.

## 2. Materials and methods

### 2.1 Preparation of the *Waltheria indica* L. plant roots

The *W. indica* plant roots were collected from the study area in the Misungwi District, Mwanza Region, Tanzania. The plant roots were carefully cleaned and then air-dried for 14 days at room temperature to prevent photo-oxidation of the active compound ingredients. Five hundred grams (500 g) of dried *W. indica* plant roots were cut into small pieces. One hundred grams (100 g) of the small pieces were ground into a coarse and fine powder using an electrical blender and stored at room temperature for further experiments. Two hundred grams (200 g) of small pieces of *W. indica* plant roots were soaked in 1 L of distilled water (27.8°C) to make a stock solution for further experiments on the disinfection of *E. coli* and *total coliforms*. Also, 200 g of *W. indica* plant roots were boiled for 8 min in 1 L distilled water to a boiling point of 100°C of water and then cooled in the refrigerator at 40°C to make a stock solution of the boiled *W. indica* plant roots (decoction method) for further experiments of disinfection of *E. coli* and *total coliforms*. The prepared liquid extracts and powdered material from the *W. indica* plant roots were finally stored in containers covered with polythene for light protection purposes and reserved for later use as a disinfectant.

### 2.2 The efficacy test of the prepared *W. indica* plant roots as a disinfectant

The disinfection efficacy of the powder, boiled, and soaked *W*. *indica* root materials against the two microbes, *E. coli* and *total coliforms,* in shallow wells of contaminated drinking water collected in the Misungwi district, as per this study, was investigated. The experiment was conducted in a randomized complete set design with four concentrations of *W. indica* plant root materials, and two controls (negative and positive controls) were included in the study. and each untreated and treated sample of water collected from shallow wells in the Misungwi district was replicated three times. Eighteen (18) beakers of 1 L of contaminated water were used, whereby twelve (12) beakers with contaminated water were disinfected with varying concentrations of powder, boiled and soaked *W. indica* plant roots, and six (6) beakers of contaminated water served as negative and positive controls. During disinfection, a varying concentration of 0 g/L, 0.2 g/L, 0.5 g/L, 0.7 g/L, and 1.0 g/L of *W. indica* powder plant roots, negative control, and positive control (0.3 mL of 1.2 mg Calcium hypochlorite) were used. For the soaked plant roots of *W. indica* stock solution, varying concentrations of 0 mL/L, 25 mL/L, 50 mL/L, 100 mL/L, and 150 mL/L were used, along with negative and positive controls, for disinfection. Additionally, in the boiled method, varying concentrations of the prepared stock solution of *W. indica* plant roots, similar to those used in the soaked method, were applied for disinfection. The treated water in the beakers was mixed thoroughly through flocculation and then swirled at 170–200 rpm for 10 minutes. It was left to settle for different time intervals of 2, 4, 6, 8, 10, 12, and 16 hours, after which the bacteriological test was conducted.

A 3M Petrifilm agar method was used for bacteriological testing after 1 mL of aliquots had been inoculated using a pipette, and sometimes MEndo agar was used, depending on the availability of agar. The 100 mL of disinfected water was prepared and filtered through 0.45 μm Millipore nitrocellulose filters using a vacuum pump. The inoculated Petrifilm and agar plates were incubated in the incubator conducted at 37°C which is a moderate temperature close to the human body temperature for *E. coli* to grow, and 44°C for *total coliforms*, which helps to selectively isolate coliforms from other bacteria like *E. coli* that may not tolerate this temperature for easy distinguishing. The incubated Petrifilm and agar plates were left for 24 hours to allow for adequate growth of both *E. coli* and *total coliforms* to detectable levels, thereby providing accurate quantification and analysis as outlined in the American Public Health Association (APHA) 2012 guidelines for procedures and methodologies for microbial water testing [25].

### 2.3 Determination of the percentage of effectiveness of disinfection methods

The percentage effectiveness in disinfection using methods of Powder, Soaked, and Boiled, based on the number of *E. coli* and *total coliform* killed, the following formula was applied after the methods’ performances had been ranked using STATISTICA software.


$$\;\text{Effective}\;\text{percentage}\;\text{for}\;\text{methods}\;\text{in}\;\text{the}\;\text{elimination}\;\text{of}\;E.\;coli=\frac{E.\;coli\;\text{in}\;\text{NC}-E.\;coli\;\text{remained}}{E.coli\;\text{in}\;\text{NC}}\times \;100\%$$ (i)

$$\begin{array}{c} \text{Effective}\;\text{percentage}\;\text{for}\;\text{methods}\;\text{in}\;\text{elimination}\;\text{of}\;Total\;coliforms\\ =\frac{Total\;coliforms\;\text{in}\;\text{NC}-Total\;coliforms\;\text{remained}}{Total\;coliforms\;\text{in}\;\text{NC}}\times 100\% \end{array}$$ (ii)

(Where NC = Negative Control)

### 2.4 Determination of pH, turbidity, and Total Dissolved Solids (TDS)

The study adhered to the standardized methods for water analysis as specified by the APHA 2012 guidelines [25]. The physicochemical parameters were assessed. Assessment of pH, turbidity, and TDS variation in untreated and treated water with plant extract as per varying concentration and contact time was done using appropriate instruments, including a portable digital pH meter (HACH HQ11D) with standardized buffer solution (base, neutral, and acid), Turbidity meter (HACH 2100Q), and EC meter which also has TDS measuring component. The results were compared with the WHO 2017 and Tanzania Bureau of Standards (TBS) 2018 guidelines, which recommend an acceptable range of 6.5 to 8.5, less than or equal to 5 NTU, and 600–1000 mg/L for pH, turbidity, and TDS in drinking water, respectively [26,27].

### 2.5 Investigation of the phytochemical compounds in the *Waltheria indica* L. (sleepy morning plant roots)

The finely powdered *roots of the* *W. indica* plant were carefully packaged and transported to the Government Chemist Laboratory Authority (GCLA) in Tanzania to identify the phytochemical compounds. This analysis was conducted using Liquid Chromatography tandem Mass Spectrometry (LC-MS/MS) equipment, whereby about 5 g of dried and powdered roots was weighed (Plate 8a) and extracted using 10 mL of methanol and then mixed by a vortex machine for solution homogeneity. The 20 mL sample of methanol was used as blank to correct errors. The sample in the falcon tube was placed in the sonicator machine for 30 minutes to facilitate extraction, then revortexed again for thorough mixing. The sample was transferred in the centrifuge device using 4000 RPM for 5 minutes. The centrifuged sample from a Falcon tube was filtered, and 1.0 mL of the resulting solution was transferred into 1.5 mL vials, which were then prepared for analysis using the LC-MS/MS equipment. This process was used to identify the bioactive chemical compounds present in the plant roots of *W. indica*.

### 2.6 Data collection

The colony-forming units (CFU) per 100 mL of the initial sample were calculated based on the number of viable colonies observed on membranes after 24 hours of incubation. The formula used to determine both *E. coli* and *total coliform* counts was:


CFU/100 mL = (Number of colonies×Dilution factor×100)/ Volume of sample filtered (in millilitres).

If no dilution was performed, the dilution factor is taken as one (1)

### 2.7 Data analysis

A Statistica tool, version 8, was used to analyse data into descriptive statistics, including the Mean and Standard error of the mean, to provide an overview of the dataset. The analysis of Variance (ANOVA) test was conducted on the data by the one-way ANOVA where the difference was significant at p < 0.05. The graphs were drawn using Excel.

## 3. Results

### 3.1 Application of the powder method of *W. indica* plant roots on the number of *E. coli*

#### 3.1.1 Concentrations of powdered *W. indica* plant roots on the number of *E. coli.*

**Fig 1:**
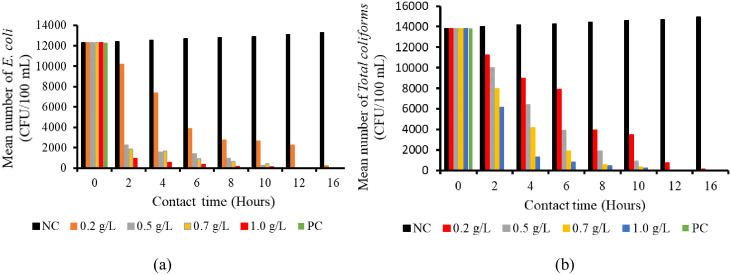
(a) Effect of concentrations on *E. coli* and (b) *total coliforms* (CFU/100 mL) with contact time.

**Fig 2:**
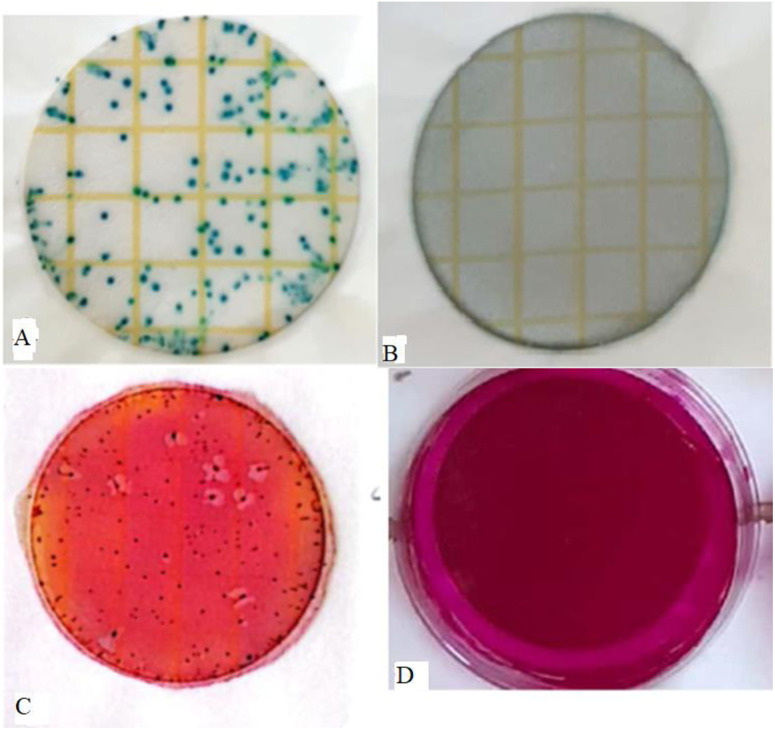
A and B show the number of *E. coli* (dark blue colonies, 12600 CFU/100 mL) and zero number of *E. coli* (0 CFU/100 mL after 12 hours) before and after application of powdered plant root materials while C and D show the number of *total coliforms* (red colonies, *> 13000 CFU/100 mL*) and zero number of *total coliform* (0 CFU/100 mL after 12 hours) before and after the application of powdered plant root materials.

Generally, the population of *E. coli* was highly and significantly (p < 0.001) reduced by higher concentrations of the *W. indica* plant root powder compared with the lower concentrations (Table 1). Additionally, the population of *E. coli* decreased significantly as the contact time increased. In contrast, a slight increase in the *E. coli* population was observed in the negative control over time (Table 1). A concentration of 0.5 g/L of *W. indica* powder eliminated *E. coli* (0.0 ± 0.0 CFU/100 mL) after 12 hours of contact time. This complete reduction was statistically equivalent (p < 0.001) to the higher concentrations of 0.7 g/L and 1.0 g/L, as well as to the positive control, all of which also showed no detectable *E. coli* (0.0 ± 0.0 CFU/100 mL). The trend of reducing of *E*. *coli* started after the application of the treatments, whereby the population of *E. coli* was reduced by application of 0.2 g/L concentration after 2 hours’ exposure and was significantly (p < 0.001) lower (10200 ± 58 CFU/100 mL) compared with the negative control (12400 ± 58 CFU/100 mL). The *E. coli* population was reduced to 233 ± 33 CFU/100 mL, lower than in the negative control (13300 ± 58 CFU/100 mL) at 16 hours. Also, the 0.5 g/L concentration of the powdered *W. indica* plant roots powder had significantly (p < 0.001) lowered (2267 ± 33 CFU/100 mL) population of *E. coli* compared with a concentration of 0.2 g/L at the contact time of 2 hours (Table 1). However, the *E. coli* were all killed at the contact time of 12 hours. The 0.7 g/L concentration of powdered plant roots showed significantly (p < 0.001) lower (1866 ± 33 CFU/100 mL) population abundance of *E. coli* compared with concentrations of 0.2 and 0.5 g/L at the contact time of 2 hours (Table 1). The *E. coli* were reduced at every 2-hour interval until 12 and 16 hours, at which point all were killed. Moreover, the 1.0 g/L concentration of powdered *W. indica* plant roots had a significantly (p < 0.001) lower (967 ± 33 CFU/100 mL) population of *E. coli* compared with concentrations of 0.2, 0.5, and 0.7 g/L at the contact time of 2 hours (Table 1). These findings suggest that the ability of the 1.0 g/L concentration of *W. indica* powdered plant roots to kill *E. coli* was enhanced by both higher concentrations and extended time of exposure (Figs 1(a), 2A and 2B).

**Table 1:** *E. coli* (CFU/100 mL) (mean ± Standard Error Mean) in response to treatment.

Concentration of Treatments (g/L)	Contact time (Hours)						
	2	4	6	8	10	12	16
0	12400 ± 58	12567 ± 33	12700 ± 58	12833 ± 33	12933 ± 33	13100 ± 33	13300 ± 58
0.2	10200 ± 58	7400 ± 33	8900 ± 58	2733 ± 33	2633 ± 33	2267 ± 33	233 ± 33
0.5	2267 ± 33	1567 ± 33	1433 ± 67	967 ± 33	433 ± 33	0 ± 0	0 ± 0
0.7	1866 ± 33	1167 ± 33	900 ± 58	667 ± 33	300 ± 58	0 ± 0	0 ± 0
1	967 ± 33	567 ± 33	400 ± 58	200 ± 0	133 ± 33	0 ± 0	0 ± 0
PC	0 ± 0	0 ± 0	0 ± 0	0 ± 0	0 ± 0	0 ± 0	0 ± 0
F-Statistic	16631.6	19676.5	10381.8	20297.9	21824	32803.29	51636.8
P-Value	<0.001	<0.001	<0.001	<0.001	<0.001	<0.001	<0.001

Each value is a mean ± standard error of three replicates, *, **, and *** significant at p ≤ 0.001.

#### 3.1.2 Efficacy of powdered *W. indica* plant roots on *total coliforms.*

The population of *total coliforms* decreased significantly (p < 0.001) in response to the concentrations of the powdered *W. indica* plant roots (Table 2). The population of *total coliforms* was significantly higher in 0.2 g/L of *W. indica* plant roots compared with 1.0 g/L at 2 hours (Table 5). Additionally, the population of *total coliforms* significantly decreased as the contact time increased, whereas a slight increase in the *total coliform* population was observed in the negative control over time (Table 2). The *total coliform* population was effectively killed (0.0 ± 0.0 CFU/100 mL) by a 0.5 g/L concentration of *W. indica* plant roots powder after a contact time of 12 hours and beyond. The effectiveness of killing the *total coliforms* was significantly (p < 0.001) the same as the 0.7 g/L, 1.0 g/L, and the positive control (0.0 ± 0.0 CFU/100 mL) after a contact time of 12 hours and beyond. The trend of reducing *total coliforms* started after the application of the treatments, whereby the population of *total coliform* was reduced by application of 0.2 g/L concentration at a contact time of 2 hours’ exposure and was significantly (p < 0.001) lower (11233 ± 33 CFU/100 mL) compared with the negative control where the number of *total coliforms* was (13833 ± 67 CFU/100 mL). The *total coliform* population decreased to 133 ± 33 CFU/100 mL at 16 hours (Table 2).

**Table 2:** *Total coliforms* (CFU/100 mL)(mean ± Standard Error Mean) in response to treatments.

Concentration of Treatment (g/L)	Contact time (Hours)						
	2	4	6	8	10	12	16
0	14000 ± 58	14167 ± 33	14300 ± 58	14433 ± 33	14600 ± 58	14700 ± 58	14933 ± 33
0.2	11233 ± 33	8967 ± 33	7900 ± 58	3967 ± 33	3500 ± 58	767 ± 33	133 ± 33
0.5	10000 ± 58	8200 ± 58	5900 ± 58	2867 ± 67	900 ± 58	0 ± 0	0 ± 0
0.7	8933 ± 33	6167 ± 33	3867 ± 67	867 ± 33	367 ± 33	0 ± 0	0 ± 0
1	8133 ± 67	1933 ± 33	1100 ± 58	667 ± 33	233 ± 33	0 ± 0	0 ± 0
PC	0 ± 0	0 ± 0	0 ± 0	0 ± 0	0 ± 0	0 ± 0	0 ± 0
F-Statistic	8839.8	16812.2	10237.62	20397.6	13094.48	37458.42	51636.8
P-Value	<0.001	<0.001	<0.001	<0.001	<0.001	<0.001	<0.001

Each value is a mean ± standard error of three replicates, *, **, and *** significant at p ≤ 0.001.

Additionally, the 0.5 g/L concentration of the powdered *W. indica* plant roots had a significantly (p < 0.001) lower population of *total coliforms* (10000 ± 58 CFU/100 mL) compared with a concentration of 0.2 g/L at a contact time of 2 hours (Table 2). However, the *total coliforms* were completely killed after contact times of 12 and 16 hours. The 0.7 g/L concentration of powdered plant roots showed significantly (p < 0.001) lower (8933 ± 33 CFU/100 mL) population abundance of *total coliforms* compared with concentrations of 0.2 and 0.5 g/L at the contact time of 2 hours (Table 2). The *total coliforms* were progressively reduced at every 2-hour interval and reached 0.0 ± 0.0 CFU/100 mL when the contact time was 12 hours and beyond. Moreover, the 1.0 g/L concentration of powdered plant roots of *W. indica* had significantly (p < 0.001) lower (8133 ± 67 CFU/100 mL) *total coliforms* compared with concentrations of 0.2, 0.5, and 0.7 g/L after a contact time of 2 hours (Table 2). This indicates that the *total coliform* killing performance of 1.0 g/L concentration of *W. indica* plant roots depends on the time interval and the tested concentrations. The effectiveness of 0.5 g/L, 0.7 g/L, and 1.0 g/L of powdered plant roots of *W. indica* in killing the *total coliform*s was significantly the same as that of a positive control (Figs 1(b), 2C and 2D)*.*

### 3.2 Application of soaked (infusion) plant materials

#### 3.2.1 Concentrations of *W. indica* plant roots on the number of *E. coli.*

**Fig 3:**
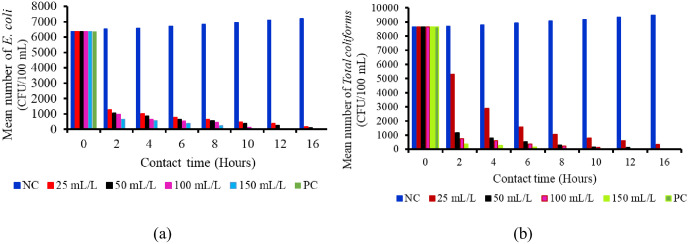
(a) and (b) show the effect of concentrations on *E. coli* and *total coliforms* (CFU/100 mL) with contact time.

**Fig 4:**
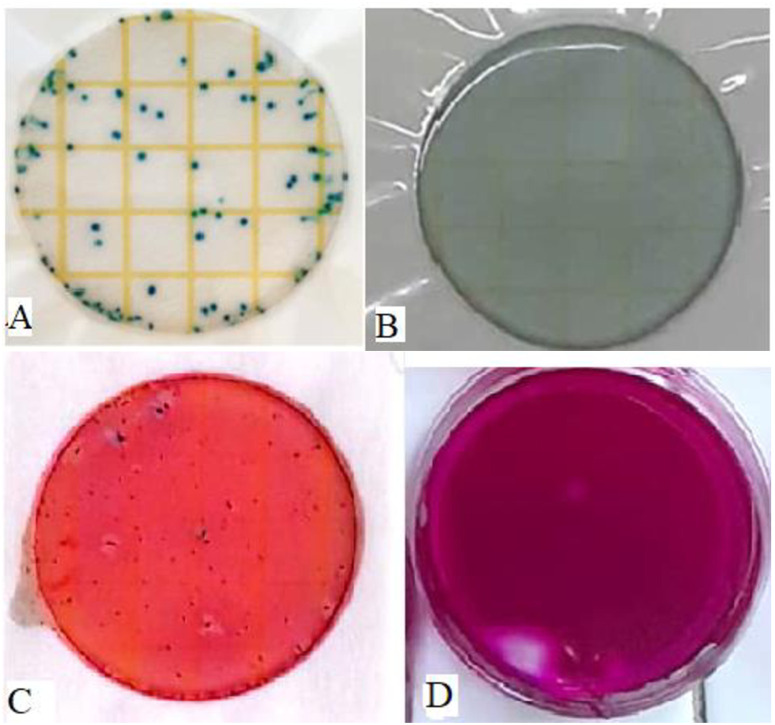
A and B show the number of *E. coli* (dark blue colonies, 6600 CFU/100 mL) and zero number of *E. coli* (0 CFU/100 mL after 12 hours) before and after application of soaked plant root extracts while C and D show the number of *total coliforms* (red colonies, *> *8700 CFU/100 mL) and zero number of *total coliform* (0 CFU/100 mL after 12 hours) before and after the application of soaked plant root extracts.

It was found that, the number of *E. coli* decreased significantly (p < 0.001) as the concentrations of the extracts of *W. indica* plant roots increased and as the contact time increased compared to the negative control whereas a slight increase of *E. coli* population was observed over time (Table 3). For instance, it was noted that only 667 ± 33 CFU/100 ml of *E. coli* remained in 150 mL/L concentrations of *W. indica* plant roots extracted through the soaked method after 2 hours of exposure. The *E. coli* was killed entirely (0.0 ± 0.0 CFU/100 mL) by 100 mL/L concentrations of *W. indica* plant roots extracted through the soaked method after a contact time of 12 hours. The effectiveness of killing the pathogen was significantly (p < 0.001) the same as the positive control (0.0 ± 0.0 CFU/100 mL) after this contact time. The trend of reducing and killing of *E*. *coli* started after the application of the treatments, whereby the number of *E. coli* was significantly (p < 0.001) lowered (1267 ± 33) compared with the negative control (6533 ± 33 CFU/100 mL) by 25 mL/L concentration of *W. indica* plant roots extracted through soaked method at a contact time of 2 hours. The decrease in the number of *E. coli* continued progressively after every two hours, whereby after 16 hours, only 167 ± 33 CFU/10mL of *E. coli* remained by using a 25 mL/L concentration of *W. indica* plant roots extracted through the soaked method. Furthermore, the 150 mL//L concentration of liquid extract (soaked *W. indica* plant roots) had significantly (p < 0.001) lower (667 ± 33 CFU/100 mL) number of *E. coli* compared with the concentrations of 25, 50, and 100 mL/L at a contact time of 2 hours (Table 3). This indicates that the *E. coli* killing and reducing performance of 150 mL/L concentration of liquid extract from soaked *W*. *indica* plant roots at time intervals was higher than other tested concentrations (Figs 3a, 4A and 4B, Table 3). A 0.3 mL dose of chlorine at a concentration of 1.2 mg/L was used as the positive control, resulting in complete elimination of *E. coli* after 30 minutes of contact time

**Table 3:** *Escherichia coli* (CFU/100 mL)(mean ± Standard Error Mean) in response to treatments.

Concentration of Treatment (mL/L)	Contact time (Hours)						
	2	4	6	8	10	12	16
0	6533 ± 33	6600 ± 58	6700 ± 58	6833 ± 33	6967 ± 33	7100 ± 58	7200 ± 58
25	1267 ± 33	1033 ± 33	767 ± 33	667 ± 33	467 ± 33	367 ± 33	167 ± 33
50	1067 ± 33	867 ± 33	667 ± 33	567 ± 33	367 ± 33	267 ± 33	100 ± 0
100	967 ± 33	667 ± 33	533 ± 33	433 ± 33	267 ± 33	0 ± 0	0 ± 0
150	667 ± 33	567 ± 33	367 ± 33	233 ± 33	133 ± 33	0 ± 0	0 ± 0
PC	0 ± 0	0 ± 0	0 ± 0	0 ± 0	0 ± 0	0 ± 0	0 ± 0
F-Statistic	5801.6	6516.4	6955.7	9741.2	10359.6	10981	13665.7
P-Value	<0.001	<0.001	<0.001	<0.001	<0.001	<0.001	<0.001

Each value is a mean ± standard error of three replicates, *, **, and *** significant at p ≤ 0.001.

#### 3.2.2 Efficacy of *W. indica* plant roots on *total coliforms.*

*Total coliforms* decreased significantly (p < 0.001) as the concentrations of the *W. indica* plant roots liquid extracted through the soaked method increased and as the contact time increased, whereas an increase of *total coliforms* population was found in the negative control as the contact time increased (Table 4). The 25 mL/L, 50 mL/L, 100 mL/L, and 150 mL//L concentrations of liquid extract (soaked *W. indica* plant roots) showed significantly (p < 0.001) lower (5300 ± 58, 1167 ± 33, 768 ± 33, 367 ± 33 CFU/100 mL, respectively) number of *total coliforms* compared with negative control (8700 ± 58 CFU/100 mL) at a contact time of 2 hours (Table 4). Furthermore, the 150 mL//L concentration of liquid extract (soaked *W. indica* plat roots) had significantly (p < 0.001) lower (367 ± 33 CFU/100 mL) number of *total coliforms* compared with the concentrations of 25, 50, and 100 mL/L at a contact time of 2 hours (Table 4). Also it was observed that *total coliforms* were completely killed (0.0 ± 0.0 CFU/100 mL) by 100 mL/L and 150 mL/L concentrations of the *W. indica* plant roots liquid extracted through the soaked method at 12 hours and 10 hours, respectively. The effectiveness of killing the pathogen was significantly (p < 0.001) the same as that of the positive control (0.0 ± 0.0 CFU/100 mL) at this contact time, and at concentrations of 100 mL/L and 150 mL/L of the *W. indica* plant root liquid extracted through the soaked method. The *total coliforms* continued to be reduced by the concentrations of plant extract every two (2) hours progressively till the number was lowest in each concentration at 16 hours of exposure (Table 4). This indicates that the *total coliforms* killing performance of 150 mL/L concentration of liquid extract from soaked *W*. *indica* plant roots at time intervals is very high compared to the other tested concentrations, since all *total coliforms* were killed at a contact time of 10 hours (Figs 3(b), 4C and 4D, Table 4).

**Table 4:** Treatment trend effect on *total coliform* (CFU/100 mL)(Mean ±Standard Error Mean) with respect to contact time.

Concentration of extract (mL/L)	Contact time (Hours)						
	2	4	6	8	10	12	16
0	8700 ± 58	8800 ± 58	8933 ± 33	9100 ± 58	9200 ± 58	9367 ± 33	9500 ± 58
25	5300 ± 58	2900 ± 58	1567 ± 33	1067 ± 33	800 ± 58	600 ± 58	333 ± 33
50	1167 ± 33	800 ± 58	533 ± 33	300 ± 58	167 ± 33	133 ± 33	0 ± 0
100	767 ± 33	600 ± 58	367 ± 33	233 ± 58	133 ± 33	0 ± 0	0 ± 0
150	367 ± 33	267 ± 33	167 ± 33	133 ± 33	0 ± 0	0 ± 0	0 ± 0
PC	0 ± 0	0 ± 0	0 ± 0	0 ± 0	0 ± 0	0 ± 0	0 ± 0
F-Statistic	9652.3	6951.0	17383.2	11014.5	12722.0	19759.4	24481.7
P-Value	<0.001	<0.001	<0.001	<0.001	<0.001	<0.001	<0.001

Each value is a mean ± standard error of three replicates, *, **, and *** significant at p ≤ 0.001.

### 3.3 Application of boiled (decoction) plant material

#### 3.3.1 Concentrations of *W. indica* plant roots on the number of *E. coli.*

It was found that the number of *E. coli* significantly decreased (p < 0.001) as both the concentration of the extracts and the contact time increased, compared to the negative control, where the *E. coli* slightly increased (Table 5). For instance, it was noted that only 1533 ± 33 CFU/100 mL of *E. coli* remained in 150 mL/L concentrations of *W*. *indica* extracted through the boiled method after 2 hours of exposure, and even at a contact time of 16 hours, the *E. coli* was not completely killed (133 ± 33 CFU/100 mL). The trend of reducing of *E*. *coli* started after the application of the treatments, whereby the number of *E. coli* was significantly (p < 0.001) lowered (4000 ± 58 CFU/100 mL) compared with the negative control (5500 ± 33 CFU/100 mL) by 25 mL/L concentration of *W. indica* plant roots extracted through boiled method at a contact time of 2 hours. The decrease in the number of *E. coli* continued progressively every two (2) hours, whereby after 16 hours, only 900 ± 58 CFU/100 mL of *E. coli* remained. Furthermore, the 150 mL//L concentration of liquid extract (boiled *W. indica* plant roots) had significantly (p < 0.001) lower (1533 ± 33 CFU/100 mL) number of *E. coli* compared with the concentrations of 25, 50, and 100 mL/L at a contact time of 2 hours (Table 5). This indicates that the *E. coli* killing and reduction in performance at a 150 mL/L concentration of liquid extract from boiled *W. indica* plant roots was higher than that of other tested concentrations. However, this concentration failed to kill all *E. coli* at a contact time of 16 hours. A 0.3 mL dose of chlorine at a concentration of 1.2 mg/L was used as the positive control, resulting in complete elimination of *E. coli* after 30 minutes of contact time.

**Table 5:** Treatment trend effect on *E. coli* (CFU/100 mL) mean ± Standard Error Mean with respect to contact time.

Concentration of Treatment(mL/L)	Contact time (Hours)						
	2	4	6	8	10	12	16
0	5500 ± 58	5600 ± 58	5733 ± 33	5867 ± 33	6000 ± 58	6133 ± 33	6200 ± 58
25	4000 ± 58	3133 ± 33	2500 ± 58	2067 ± 33	1567 ± 33	1367 ± 33	900 ± 58
50	3433 ± 33	2400 ± 58	1967 ± 33	1567 ± 33	1167 ± 33	867 ± 33	733 ± 33
100	2100 ± 58	1567 ± 33	967 ± 33	667 ± 33	567 ± 33	367 ± 33	267 ± 33
150	1533 ± 33	1067 ± 33	733 ± 33	367 ± 33	267 ± 33	167 ± 33	133 ± 33
PC	0 ± 0	0 ± 0	0 ± 0	0 ± 0	0 ± 0	0 ± 0	0 ± 0
F-Statistic	2176.6	2838.5	4025.6	6021.3	4876.1	7021.6	4436.8
P-Value	<0.001	<0.001	<0.001	<0.001	<0.001	<0.001	<0.001

Each value is a mean ± standard error of three replicates, *, **, and *** significant at p ≤ 0.001.

#### 3.3.2 Efficacy of boiled *W. indica* plant roots on *total coliforms.*

Also, the number of *total coliforms* significantly decreased (p < 0.001) as both the concentration of the boiled extracts and the contact time increased, compared to the negative control (Table 6). The 25 mL/L, 50 mL/L, 100 mL/L, and 150 mL/L concentrations of liquid extract (boiled *W. indica* roots) showed significantly (p < 0.001) lower (6800 ± 58, 5000 ± 33, 4133 ± 33, 2067 ± 33 CFU/100 ml, respectively) number of *total coliforms* compared with negative control (8600 ± 58 CFU/100 mL) at a contact time of 2 hours (Table 6). Furthermore, the 150 mL/L concentration of liquid extract (boiled *W. indica* roots) had significantly (p < 0.001) lower (2067 ± 33 CFU/100 mL) number of *total coliforms* compared with the concentrations of 25, 50 and 100 mL/L at a contact time of 2 hours (Table 6). It was observed that the number of *total coliforms* was completely killed (0.0 ± 0.0) by 50 mL/L, 100 mL/L, and 150 mL/L concentrations of the *W. indica* liquid extracted through the boiled method at 16 hours, 12 hours, and 10 hours, respectively. The effectiveness of killing the pathogen was significantly (p < 0.001) the same as that of the positive control (0.0 ± 0.0) at this contact time, using 50 mL/L, 100 mL/L, and 150 mL/L concentrations of the *W. indica* liquid extracted through the boiled method. The *total coliforms* continued to be reduced by the concentrations of plant extract every two (2) hours, progressively until the lowest number was reached in each concentration at 16 hours of exposure (Table 6). This indicates that the *total coliform* killing performance of the extract from boiled *W*. *indica* plant roots increased as the tested concentration and contact time increased.

**Table 6:** Treatment trend effect on *total coliforms* (CFU/100 mL) mean ± Standard Error Mean with respect to contact time.

Concentration of Treatment(mL/L)	Contact time (Hours)						
	2	4	6	8	10	12	16
0	8600 ± 58	8700 ± 33	8833 ± 33	9000 ± 58	9167 ± 33	9233 ± 33	9400 ± 58
25	6800 ± 58	4833 ± 52	3600 ± 58	2500 ± 58	1633 ± 33	767 ± 33	500 ± 58
50	5000 ± 58	2700 ± 58	1167 ± 33	767 ± 33	567 ± 33	267 ± 33	0 ± 0
100	4133 ± 33	1767 ± 33	933 ± 33	633 ± 33	367 ± 33	0 ± 0	0 ± 0
150	2067 ± 33	867 ± 33	533 ± 33	433 ± 33	233 ± 33	0 ± 0	0 ± 0
PC	0 ± 0	0 ± 0	0 ± 0	0 ± 0	0 ± 0	0 ± 0	0 ± 0
F-Statistic	5536.4	7989.3	11432.1	9796.4	17563.4	28515.5	16992.1
P-Value	<0.001	<0.001	<0.001	<0.001	<0.001	<0.001	<0.001

Each value is a mean ± standard error of three replicates, *, **, and *** significant at p ≤ 0.001.

## 4. Disinfection effectiveness of powder, boiled, and soaked extracts

The effectiveness of powder, boiled, and soaked extract methods for disinfecting shallow well water was analysed in terms of *Escherichia coli* and *total coliform* elimination at a contact time of 16 hours.

**Fig 5:**
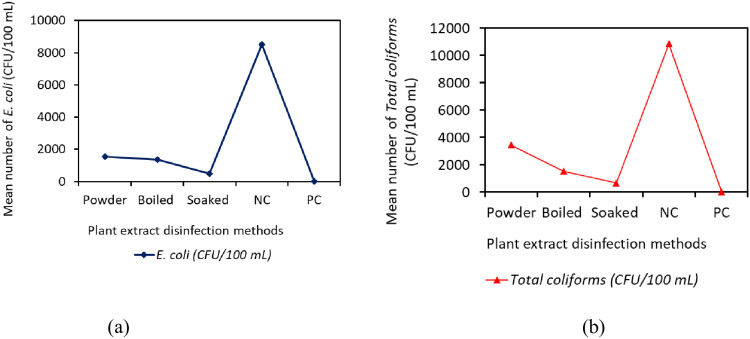
(a) and (b) show the effectiveness of powder, boiled, and soaked methods in eliminating *E. coli* and *total coliforms* in 16 hours.

Generally, the study found that the soaked extract significantly (p < 0.001) reduced the *E. coli* (481 ± 38 CFU/100 mL) by 94.3% compared to both the boiled extract (1373 ± 111 CFU/100 mL) by 83.9% and the powder form (1543 ± 250 CFU/100 mL) by 81.9%. In contrast, the number of *E. coli* in the negative control was 8514 ± 393 CFU/100 mL after 16 hours. The results indicate that the soaked method was the most effective for eliminating *E. coli,* achieving a 94.3% killing efficiency. This was followed by the boiled method, which reduced *E. coli* by 83.9%, and the powder method, which achieved an 81.9% reduction efficiency (Table 7 and Fig 5(a)).

**Table 7:** Effective percentage for methods in elimination of *E. coli* and *total coliforms.*

Microbes	Killing effectiveness (%)	
disinfection method	*E. coli*	*Total coliforms*
Powdered *W. indica* plant roots	81.9	68.2
Soaked *W. indica* extract plant roots	94.3	93.8
Boiled *W. indica* extract plant roots	83.9	85.8

Also, the soaked extract continued to dominate in the elimination of *total coliforms* significantly (p < 0.001, 668 ± 118 CFU/100 mL) by 93.8% reduction efficiency compared to the boiled (1538 ± 195 CFU/100 mL, 85.8%) and lastly powder form (3450 ± 405 CFU/100 mL) by 68.2%, referenced to negative control (10841 ± 326 CFU/100 mL) and positive control (0 ± 0 CFU/100 mL) as shown in Table 7 and Fig 5b. The results indicate that the soaked method was the most effective for eliminating *total coliforms,* achieving a 93.8% reduction. This was followed by the boiled method, which reduced *total coliforms* by 85.8%, and the powder method, which achieved a 68.2% reduction (Table 7 and Fig 5(b)).

### 4.1 Levels of pH in disinfected shallow well water using powder, soaked, and boiled methods

**Fig 6:**
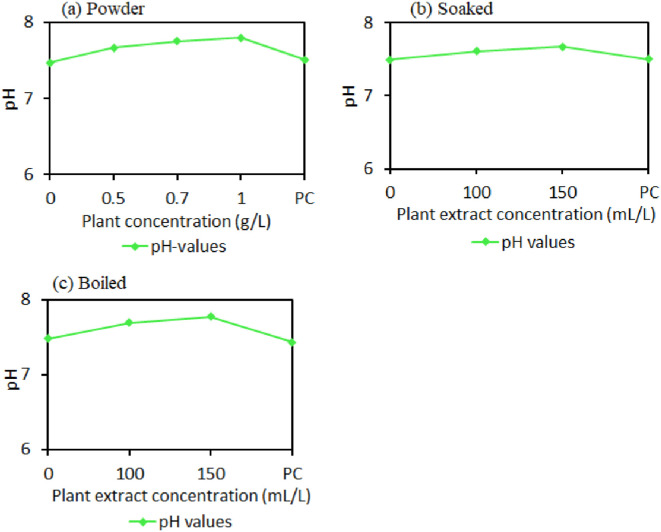
(a), (b), and (c) shows the variations in pH of well water samples treated with different concentrations in powder, soaked, and boiled forms at a 12-hour contact time.

The pH level with different concentrations of powder, boiled, and soaked plant extract form was determined to represent the mortality range of *E. coli* and *total coliforms* at a contact time of 12 hours of shallow well water disinfection. A slight increase in pH level with an increase in extract concentration was noted compared to raw water pH for powder, soaked, and boiled extract methods (Fig 6(a)–6(c)). However, the pH level stayed within the acceptable range as per the WHO 2017 guideline, which recommends that the pH of drinking water should be between 6.5 and 8.5 (Fig 6(a)–6(c)).

### 4.2 Turbidity in disinfected shallow well water using powder, soaked, and boiled methods

**Fig 7:**
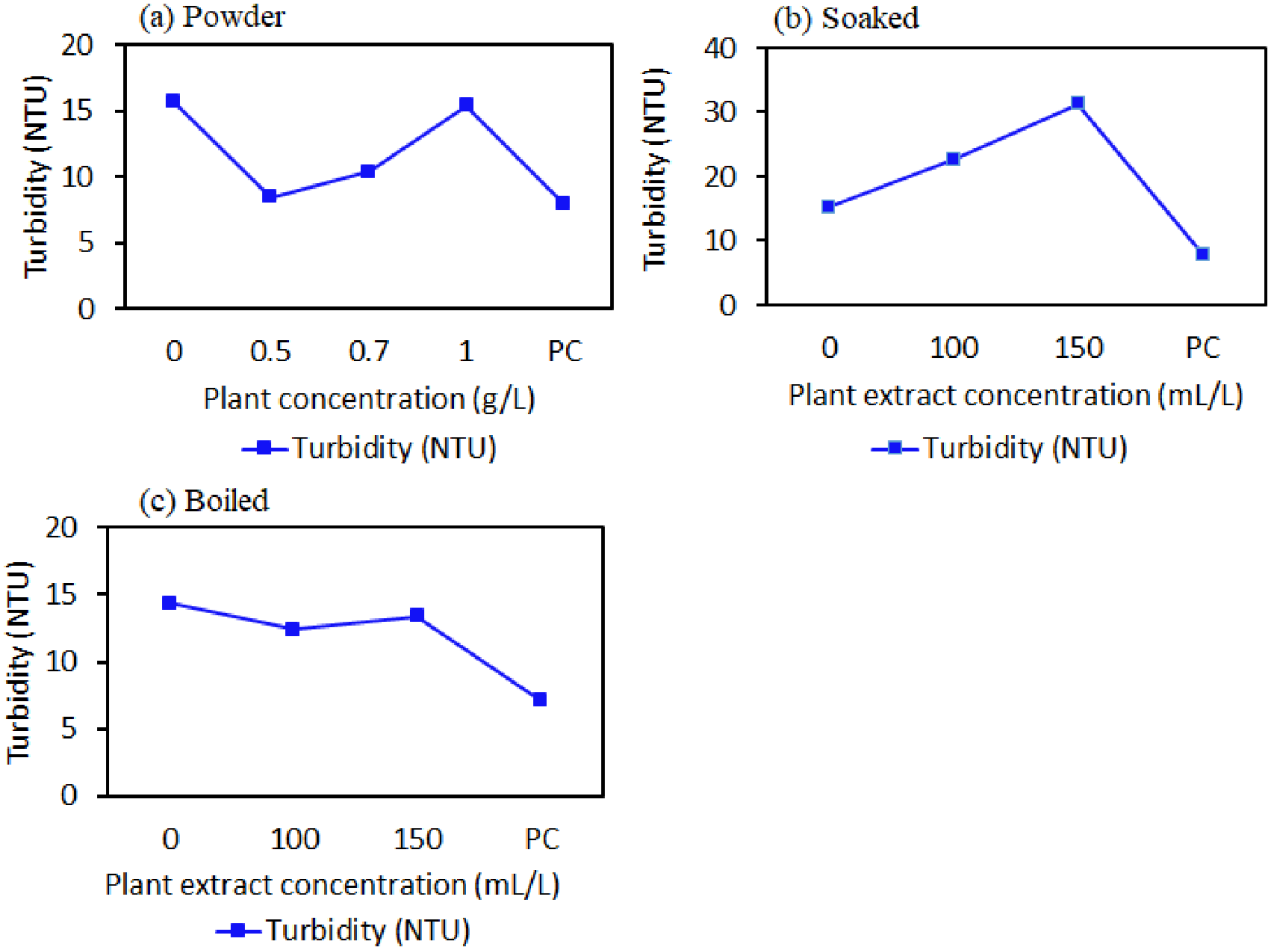
(a), (b), and (c) shows the variations in Turbidity of well water samples treated with different concentrations of powder, soaked, and boiled forms at a 12-hour contact period.

**Fig 8:**
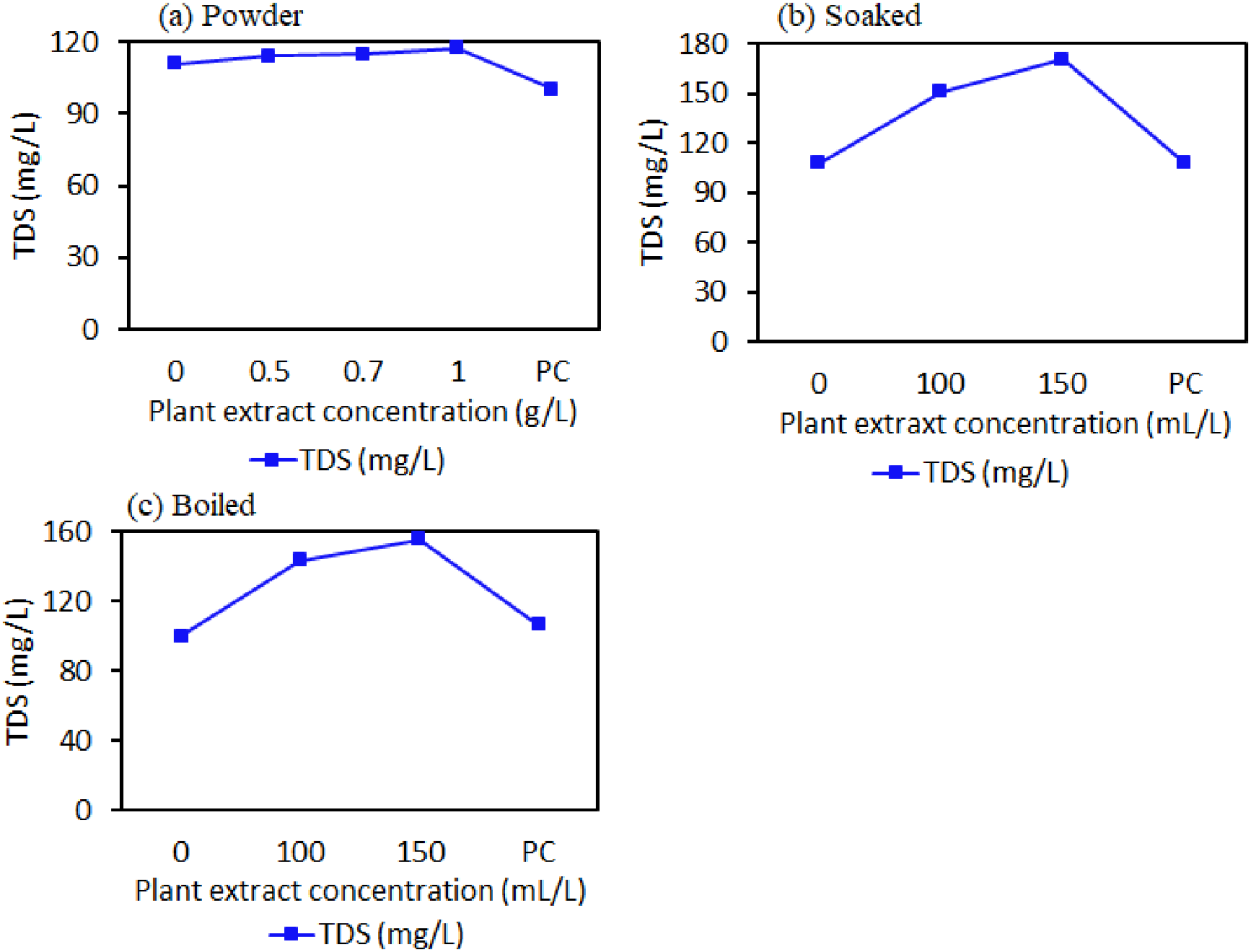
(a), (b), and (c) shows the variations in TDS of well water samples treated with different concentrations of powder, soaked, and boiled forms at a 12-hour contact period.

The turbidity level was also determined in the treated water to show if there was any significant change in relation to the different concentrations of powder, boiled, and soaked plant extract materials after 12 hours of disinfection. It was found that there was a significant (p < 0.001) decrease in turbidity level with powder and boiled plant extract for disinfection (Fig 7(a) and 7(c)) compared to raw water from 15.73 ± 0.07 to 8.43 ± 0.006 NTU and from 14.40 ± 0.003 NTU to 12.41 ± 0.006 NTU. In contrast, an increase in turbidity level for the soaked extract method was also found compared to raw water from 15.30 ± 0.006 NTU to 22.64 ± 0.023 NTU by 48.0% and from 15.30 ± 0.006 NTU to 31.28 ± 0.042 NTU by 104.0% at the concentration of 100 mL/L and 150 mL/L (Fig 8b). Additionally, the turbidity level for positive control (PC) was significantly lower (7.91 ± 0.003, 7.87 ± 0.006 and 7.12 ± 0.006 NTU) compared to the turbidity levels after treatment using plant material in powder, soaked and boiled forms, respectively. However, the turbidity level exceeded the WHO 2017 guideline value of 5 NTU. Research on turbidity removal is currently in progress.

### 4.3 TDS levels in disinfected shallow well water using powder, soaked and boiled methods

Total Dissolved Solids (TDS) were determined in the treated water with different concentrations of powder, soaked, and boiled *W. indica* plant root materials to assess their effects on TDS after 12 hours of contact time in shallow well water treatment. There was a slight increase of TDS for the disinfected water through powder, soaked and boiled extract of *W. indica* plant root materials compared to raw water (111.1 ± 0.07, 107.7 ± 0.03 and 100.1 ± 0.03 mg/L and positive control (PC) 100.3 ± 0.03, 107.8 ± 0.06 and 106.2 ± 0.03 respectively as shown in Fig 8(a)–8(c). In the powder method, there was a slight increase of TDS for the concentration of 0.5, 0.7 and 1.0 g/L (Fig 8a), which could be accounted for by powdered plant material not fully dissolved in the water from the shallow well, attracting the accumulation of suspended particles. However, the shallow wells disinfected water using powder, boiled, and soaked extract methods exhibited lower TDS levels, which fall within the acceptable limit of 600 mg/L as recommended by the WHO 2017 guidelines for drinking water.

### 4.4 Bioactive chemical composition of *Waltheria indica* plant roots relevant to pathogens elimination

**Fig 9:**
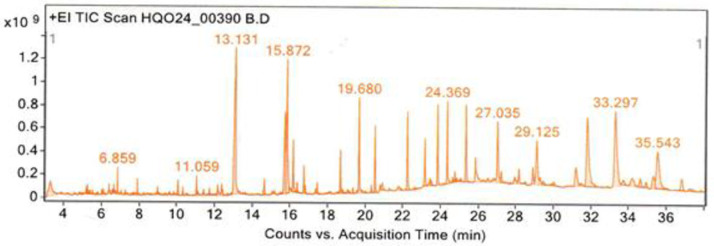
Chromatogram showing peak lists of identified phytoconstituents of *W. indica* plant roots.

The LC-MS/MS results identified various phytochemical compounds in the LC-MS/MS fractions of methanolic extraction of powdered *W. indica* plant roots. The chromatogram of the detected compounds from the selected plant root extracts was compared with the spectra of the known compounds stored in the National Institute of Standards and Technology (NIST) library. The name, retention time, molecular weight and molecular formula of the compounds contained in *W. indica* plant root extracts are presented in Fig 9 and Table 8. The phytochemical compounds identified were vanillin, phenol,2,5-bis(1,1-dymethylethyl), neophytadiene, 2,2-Dihydroxychalcone, phenol,2,2-methylenebis[6-(1,1-dimethylethyl)-4-methyl, squalene, hexadecanamide, lupeol, 9-Octadecanamide (Z) and phenol,2,4-bis(1,1-dimethyl)-phosphite (3:1), which are thought to be effective in pathogen elimination from shallow well water.

**Table 8:** Organic compounds with antibacterial activity obtained from the methanolic root extract of *W. indica* plant detected by LC-MS/MS.

Retention time(min)	Compound name	Molecular formula	Molecular weight (g/mol)	References
6.098	Vanillin	C_8_H_5_O_2_	133.12	[28]
6.859	Phenol,2,5-bis(1,1-dymethylethyl)	C_14_H_22_	190.32	[29]
11.059	Neophytadiene	C_5_H_6_	66.10	[30]
15.872	2,2-Dihydroxychalcone	C_15_H_12_O_3_	240.08	[31]
19.680	Phenol,2,2-methylenebis[6-(1,1-dimethylethyl)-4-methyl	C_9_H_19_O_2_	159.25	[32]
24.369	Squalene	C_4_H_10_	58.12	[33]
27.035	Hexadecanamide	C_16_H_33_NO	255.44	[34]
35.543	Lupeol	C_12_H_22_	166.30	[35]
29.125	9-Octadecanamide (Z)	C_18_H_35_NO	281.48	[36]
36.018	Phenol,2,4-bis(1,1- dimethyl)-phosphite (3:1)	C_31_H_35_O	423.61	[37]

## 5. Discussion

The use of dried *W. indica* plant roots in different forms (powdered, soaked and boiled) has been considered in this study for the disinfection of *Escherichia coli* and *total coliforms* in shallow well water. Among these methods, the soaked extract demonstrated the most effective reduction of *E. coli* and *total coliforms*, achieving 94.3% and 93.8% removal. The boiled method reduced *E. coli* by 83.9% and *total coliforms* by 85.8%, while the powdered form resulted in 81.9% and 68.2% reductions of *E. coli* and *total coliforms.* The superior performance of the soaked extract is likely attributed to the preservation of volatile phytochemical compounds, which are known to possess potent antimicrobial properties. This observation aligns with findings by [38] who reported that soaking plant materials allows for better retention of heat-sensitive bioactive compounds, enhancing their antimicrobial potential in water purification. Similarly, [39] demonstrated that soaking *Moringa oleifera* seeds led to improved bacterial reduction in water compared to thermal processing methods. The reduced efficacy observed in the boiled extract, particularly when boiled for extended durations (8 and 15 minutes at 100°C), may result from the degradation of heat-sensitive phytoconstituents such as flavonoids, terpenoids and phenolic compounds. This finding is consistent with [40], who reported that excessive boiling of plant materials can diminish their antibacterial effectiveness by degrading volatile bioactive compounds. Consequently, for the powdered form of *W. indica* plant roots, although antibacterial activity was observed, its slower disinfection process can be explained by the interaction of active compounds with plant fibres, limiting their availability for microbial inactivation. This explanation aligns with the work of [41], who reported that finer particle sizes of plant powders enhance the contact surface area between the extract and pathogens, improving disinfection efficiency. Moreover, the use of *W.indica* plant root extracts across various concentrations effectively reduced microbial contamination without significantly altering Total Dissolved Solids (TDS) and pH levels, maintaining compliance with WHO 2017 and TBS 2018 drinking water guidelines as enforced by the Energy and Water Utilities Regulatory Authority (EWURA) [42]. Similar outcomes were reported by [43], who found that plant-based water treatments provided significant reductions in pathogen levels while maintaining acceptable physicochemical water quality. The consistent decrease in the number of *E. coli* and *total coliforms* in treated water over time confirms the efficacy of *W. indica* plant root extracts. Conversely, the slight increase of *E. coli* and *total coliforms* in the negative control likely results from availability of trace nutrients such as organic or inorganic phosphorous, which support massive growth and proliferations. A trend noted in earlier studies by [44] regarding nutrient enriched untreated water.

The reduction of pathogens can be attributed to various phytoconstituents from the *W. indica* plant roots, identified in this study through LC-MS/MS analysis, including vanillin, neophytadiene, flavonoids (e.g., 2,2-Dihydroxychalcone), squalene, lupeol, and terpenes. These compounds are well-documented for their broad-spectrum antibacterial properties. For instance, [33] and [45] emphasized the role of flavonoids and terpenes in disrupting bacterial cell membranes, which leads to cell death. At the same time, [35] highlighted the efficacy of lupeol and squalene in the development of antibacterial agents targeting gastrointestinal pathogens. These findings further support previous studies by [46] and [47], who reported the successful application of plant-derived phytochemicals for water disinfection, especially in rural communities lacking access to conventional water treatment infrastructure. Furthermore, the phytochemical analysis using LC-MS/MS revealed the presence of phenol, 2,5-bis(1,1-dimethylethyl), phenol,2,2-methylenebis[6-(1,1-dimethylethyl)-4-methyl], hexadecanamide, 9-octadecenamide (Z) and phenol,2,4-bis(1,1-dimethyl)-phosphite (3:1) of *W. indica* plant roots which can be thought to eliminates pathogens. These findings are consistent with those documented by [29,32,34], who highlighted the efficacy of the antibacterial properties of such compounds. The presence of these bioactive constituents likely contributed to the observed elimination of *E. coli* and *total coliforms* from shallow well water, thereby improving its safety for drinking and other domestic uses.

## 6. Conclusion

The findings of this study demonstrate that plant-based extracts from *Waltheria indica* plant roots offer a promising alternative for disinfecting shallow well water, particularly in resource-limited settings. The three tested forms (powdered, soaked, and boiled) resulted in a significant reduction of *E. coli* and *total coliforms,* with efficacy increasing alongside both extract concentration and contact time. Complete elimination of both *E. coli* and *total coliforms* was achieved after 12 hours using 0.5 g/L of powdered roots and 100 mL/L of soaked material, while the boiled extract (100 mL/L) successfully eliminated only *total coliforms* within the same timeframe. The antibacterial potential of *W. indica* plant roots is supported by the presence of key phytochemical compounds identified through LC-MS/MS analysis, including vanillin, neophytadiene, lupeol, squalene, and various phenolic derivatives, all of which are known for their antimicrobial properties. These compounds may play a crucial role in enhancing the safety of shallow well water, particularly in rural Tanzania and other developing regions. Overall, this research contributes valuable knowledge to the field of natural water treatment options, providing evidence for *W. indica* plant root extracts as an effective, plant-based alternative for improving drinking water quality. The outcomes are expected to support efforts toward bridging knowledge gaps in water treatment and advancing access to safe water in underserved communities.
